# Max Lewandowsky (1876–1918)

**DOI:** 10.1007/s00415-019-09393-y

**Published:** 2019-05-27

**Authors:** Filip Marcinowski

**Affiliations:** Department of Psychiatry, Medical University of Warsaw, Nowowiejska 27, 00-665 Warsaw, Poland

In the first two decades of the twentieth century, there was probably no European neurologist who was not familiar with the name of Max Lewandowsky, German-Jewish neurologist, author of numerous works, including a handbook of neurology, and the editor of the neurological journal *Zeitschrift für die gesamte Neurologie und Psychiatrie*. Today, he is rarely remembered, except mostly in the context of his research on the blood–brain barrier.

**Fig. 1:**
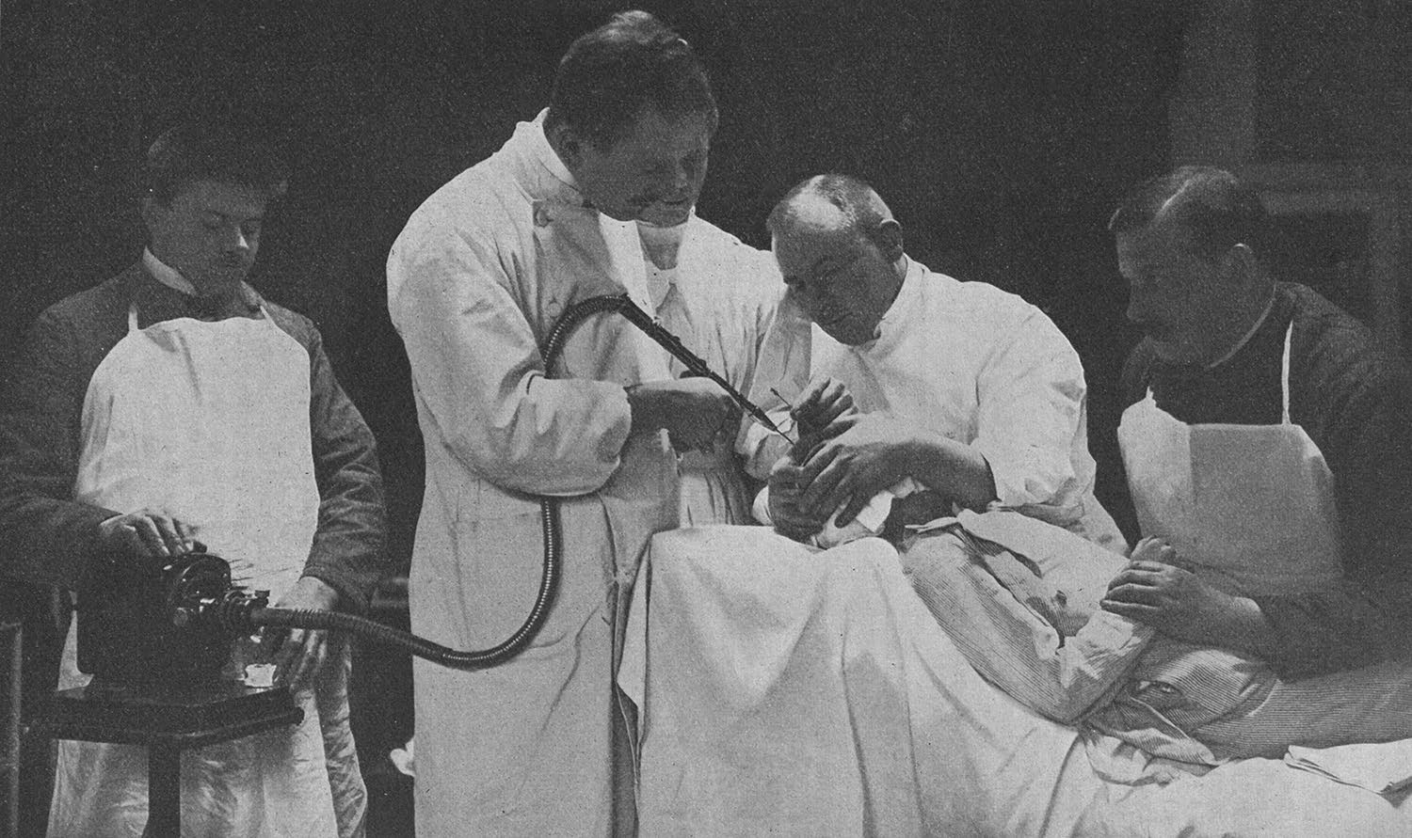
Max Lewandowsky (second from the left) performing a brain puncture, from [2, Bd 1, p 1192]

Max Heinrich Lewandowsky was born on June 28, 1876 in Berlin, the son of Hermann Lewandowsky and Rose née Heymann. He attended *Friedrichsgymnasium* in Berlin, graduating in 1893. Then he studied medicine in Marburg, Berlin and Halle. Among his teachers were Theodor Engelmann (1843–1909) and Hermann Munk (1839–1912). He defended his Ph.D. thesis in 1898 and immediately continued his scientific career, first in the physiology laboratory in Berlin. He attended courses of psychiatry led by Karl Bonhoeffer (1868–1948) and Franz Nissl (1860–1919) in Heidelberg and Theodor Ziehen in Berlin Charité clinic. In Paris he studied under Pierre Marie (1853–1940) at Bicêtre Hospital. In 1902 he became Privatdozent in Berlin, and in 1908 he was appointed extraordinary professor [1]. Further scientific career progress was unattainable for him because of his Jewish descent (Fig. 1).

Lewandowsky was an efficient organizer. In 1910, together with Alois Alzheimer (1864–1915), he founded *Zeitschrift für die gesamte Neurologie und Psychiatrie*. It was accompanied ‘by a publishing series “Monographs from the joint field of neurology and psychiatry” (*Monographien aus dem Gesamtgebiete der Neurologie und Psychiatrie*). In the same year, he began work on a multi-authored handbook of neurology, inviting dozens of renowned specialists from Germany and abroad [2]. Until 1915, five volumes of this book were published, bringing him well-deserved recognition. Today, Lewandowsky's handbook is credited as the first venture of this kind [3]. Lewandowsky himself was an author of more than 20 chapters of this work, covering such topics as the anatomy of the sympathetic system, general physiology of the central nervous system, ataxia, brain trauma, brain abscesses, psychiatric disorders, myasthenia, tetanus, hysteria etc., which give an overview of his broad scientific interests.

As a researcher, Lewandowsky was particularly interested in experimental work. His Ph.D. dissertation dealt with vagal control of lung function [4]. Under the supervision of Oskar Vogt (1870–1959), he investigated brainstem pathways. Lewandowsky continued Paul Ehrlich’s (1854–1915) experiments on the diffusion of intravenously administered dyes to the central nervous system, which led him to introduce the term of blood–brain barrier (*Bluthirnschranke*) [5]. His subsequent works on the pharmacotherapy of the central nervous system were interrupted by World War I and his untimely death [1]. In 1907, Lewandowsky published a handbook of the functions of the central nervous system [6]. He also authored a well-received handbook of neurology for practising physicians, which ran to three editions [7].

Max Lewandowsky was married to a mezzosoprano singer Margarete (Gretchen) Gille in 1909; the marriage produced no children. After World War I broke out, Lewandowsky served as an army physician. He investigated neurological symptoms in head trauma and opposed inhuman methods of treating war neuroses [8]. In the summer of 1917, he was sent to the western front and to the Balkans, where he contracted typhoid fever and was admitted to Bucharest hospital [9]. He ended his life in a private sanatorium Reserve Lazarett Haus Schönow in Berlin-Zehlendorf, where he was hospitalized with post-typhoid depression [10]. He committed suicide by cutting his wrists and stabbing his heart. He was buried in a Jewish cemetery in Berlin-Weißensee. Lewandowsky's death received much attention in the neurological press, but its circumstances were taboo, just as in the case of Max Rothmann (1868–1915), another German-Jewish neurologist who took his own life during the war.
